# Practice of Medicine

**Published:** 1895-01

**Authors:** 


THE PRACTICE OF MEDICINE, A SYNOPSIS OF. Comprising an embodiment of the late "Systems" and Cyclopædias. By Wm. B. Stewart, M. D., of Philadelphia. 433 pages. $2.75.

